# Co-Expression of Bacterial Aspartate Kinase and Adenylylsulfate Reductase Genes Substantially Increases Sulfur Amino Acid Levels in Transgenic Alfalfa (*Medicago sativa* L.)

**DOI:** 10.1371/journal.pone.0088310

**Published:** 2014-02-10

**Authors:** Zongyong Tong, Can Xie, Lei Ma, Liping Liu, Yongsheng Jin, Jiangli Dong, Tao Wang

**Affiliations:** State Key Laboratory of Agrobiotechnology, College of Biological Sciences, China Agricultural University, Beijing, China; Leibniz-Institute for Vegetable and Ornamental Plants, Germany

## Abstract

Alfalfa (*Medicago sativa* L.) is one of the most important forage crops used to feed livestock, such as cattle and sheep, and the sulfur amino acid (SAA) content of alfalfa is used as an index of its nutritional value. Aspartate kinase (AK) catalyzes the phosphorylation of aspartate to Asp-phosphate, the first step in the aspartate family biosynthesis pathway, and adenylylsulfate reductase (APR) catalyzes the conversion of activated sulfate to sulfite, providing reduced sulfur for the synthesis of cysteine, methionine, and other essential metabolites and secondary compounds. To reduce the feedback inhibition of other metabolites, we cloned bacterial *AK* and *APR* genes, modified *AK*, and introduced them into alfalfa. Compared to the wild-type alfalfa, the content of cysteine increased by 30% and that of methionine increased substantially by 60%. In addition, a substantial increase in the abundance of essential amino acids (EAAs), such as aspartate and lysine, was found. The results also indicated a close connection between amino acid metabolism and the tricarboxylic acid (TCA) cycle. The total amino acid content and the forage biomass tested showed no significant changes in the transgenic plants. This approach provides a new method for increasing SAAs and allows for the development of new genetically modified crops with enhanced nutritional value.

## Introduction

Alfalfa (*Medicago sativa* L.) is one of the most important forage crops and is grown throughout the world. As fodder, this crop provides proteins, vitamins, and other minerals to such ruminants as cattle and sheep. Due to the lack of a biosynthetic pathway for methionine, cysteine, lysine, and other essential amino acids (EAAs) *in vivo*, animals must obtain these EAAs from their diet. Sulfur amino acids (SAAs), mainly methionine and cysteine, are particularly important EAAs, and previous studies have recognized the importance of sulfur in alfalfa [1]. The effects of amino acids in ruminants have been studied [2], and it has been reported that low amounts of SAAs in forage limit wool growth in sheep and milk production and meat quality in cattle [3], [4]. Indeed, quality breeding, particularly to increase the content of SAAs, is the most important aspect of alfalfa engineering.

Compared to traditional breeding methods, transgenic techniques are more efficient for directly manipulating the characteristics of plants, and the transfer of a few sulfur-rich protein genes into plants has been reported using engineering technology [5]–[7]. The SAA levels in transgenic alfalfa were increased via the transformation of the *sunflower seed albumin* (*SSA*) gene, which led to the production of an additional 40 mg of SAAs in the leaves; when these leaves were fed daily to sheep, their wool growth rates were significantly increased [5]. It was also reported that overexpressing sulfur amino acid-rich protein (HNP) [6] and *Arabidopsis* cystathionine γ-synthase (*At*CGS) [7] could significantly increase SAAs in transgenic alfalfa. Although this method has led to some success, most of these high-SAA proteins were found to be unstable in the plants [8]–[10]; furthermore, the increase in SAA in the transgenic plants occurred at the expense of other endogenous sulfur-rich proteins or compounds [11], [12].

There is an increasing demand for high-protein forage due to the rapid development of the animal industry, and there is a constant need for more effective genes that can enhance desirable characteristics in livestock. Aspartate kinase (AK) catalyzes the phosphorylation of aspartate, forming Asp-phosphate. This phosphorylation is the first step in the aspartate family biosynthesis pathway and is regulated by several feedback inhibition loops in plants [13]. Threonine feedback inhibits the activity of AK-homoserine dehydrogenase [14], whereas lysine negatively regulates the activity of the monofunctional AK isozymes [6], [15], [16]. However, site-directed mutagenesis of *AKIII* from *Escherichia coli* was performed to substitute an isoleucine for a threonine at amino acid 352, which reduced the feedback inhibition of *AK* by lysine [17]. Overexpression of a bacterial feedback-insensitive AK in transgenic plants led to an increase in threonine that was accompanied by a reduction in both aspartate and glutamate; whereas the level of methionine, which diverges from this branch, was not significantly altered [18], [19]. Additionally, the seed-specific expression of a bacterial AK was shown to increase the threonine and methionine levels in the seeds of transgenic tobacco [20].

Because overexpressing AK can increase the level of Asp family amino acids, particularly threonine, in transgenic alfalfa [19], the sulfur metabolic pathway might be useful for increasing the content of cysteine and methionine. Therefore, we focused on the key enzyme in the plant sulfate assimilation pathway: 5'-adenylylsulfate reductase (APR). Sulfate assimilation provides reduced sulfur for the synthesis of cysteine, methionine, and other essential metabolites and secondary compounds, and the conversion of sulfate to sulfite, which is catalyzed by APR, is considered to be the key step in sulfate assimilation in higher plants [21]–[23]. Plant APR is dependent on reduced glutathione as the electron donor [24], [25], whereas bacterial APR requires thioredoxin or glutaredoxin as reductants [26]–[29]. However, bacterial APR also can utilize plant thioredoxins [28], [30], [31]; in fact, the overexpression of a bacterial APR resulted in the accumulation of cysteine in *Zea mays* [32]. *Pseudomonas aeruginosa* APR (*Pa*APR) is a highly stable enzyme, and, unlike the endogenous APR in plants, *Pa*APR is feedback insensitive to its downstream products. The overexpression of *Pa*APR in *Arabidopsis thaliana* led to a 1.5-2-fold increase in sulfur compounds, with inorganic sulfite and thiosulfate increasing more than cystathionine γ-synthase (CGS) and glutathione [33].

Co-expressing two or more genes can enhance the targeted characteristics of transgenic plants. In general, co-expression can be achieved by either crossing two different transgenic plants or constructing an expression vector containing two different gene cassettes for subsequent plant transformation; the second method is more convenient, stable, and effective and less time-consuming. In cross breeding, a gene can easily segregate in the offspring; however, a co-expressing vector can integrate into a chromosome and be stably inherited by the progeny. By crossing two transgenic tobacco lines, one overexpressing the feedback-insensitive bacterial enzyme DHPS and the other overexpressing *At*CGS, enhanced levels of methionine and its metabolite, S-methylmethionine (SMM), were obtained [34]. Transgenic plants obtained by crossing transgenic tobacco plants overexpressing the bacterial *AK* gene with plants overexpressing *At*CGS have been shown to have significantly higher methionine and threonine levels [13]. However, the means by which alfalfa can be transformed using a vector containing two or more genes to increase SAAs remains unclear.

As the synthesis of aspartate family amino acids and the sulfate assimilation process occur in the chloroplast [33], [35], [36], we ligated a chloroplast transit peptide to modified-AK and APR to localize these enzymes to the chloroplasts. We then transformed alfalfa with a vector containing both of the genes for these enzymes to increase the content of SAAs and modify the nutritional value of alfalfa. The results revealed a substantial increase in SAAs in the transgenic alfalfa; other EAAs, such as Lys and Asp, were increased as well.

## Materials and Methods

### Ethic Statement

All animal procedures were approved by the Institutional Animal Care and Use Committee at China Agricultural University. All surgery was performed under sodium pentobarbital anesthesia, and all efforts were made to minimize suffering.

### Vector construction

To reduce feedback inhibition from downstream metabolites, we modified the nucleotide sequence of *AKIII* from *E. coli* strain *K12* (GenBank accession: NC_000913.2) via overlap extension PCR [37]. The primer pairs used for mutating *AKIII* are listed in Table S1. The first-round PCRs involved two primer pairs: AK-fr/AKmu-rv and AKmu-fr/AK-rv. The two first-round PCR products were mixed and utilized as the template for the second round of PCR using the primer pair: AK-fr/AK-rv. Through site-directed mutagenesis, a T was substituted for the C at nucleotide position 1055 in *AKIII*; thus, isoleucine was substituted for threonine at amino acid position 352. The *APR* gene (GenBank accession: NC_250447.1) was amplified from *P. aeruginosa* strain PAO1 using the primer pair: APR-fr/ APR-rv. The gene sequences were confirmed by sequencing.

The Recombination-assisted Multifunctional DNA Assembly Platform (RMDAP) [38] contains 14 pairs of satellite vectors (pOSB series) and three types of recipient vectors. We chose pOSB108 and pOSB208, which both contain a chloroplast transit peptide, as the satellite vectors. pDES200 containing the *NPTII* gene was used as the recipient vector, and *E. coli* strain SW106, which contains all the requisite *in vivo* recombination proteins, including an arabinose-inducible *cre* gene, was used as the recipient host. *AK* was subcloned into pOSB108, and *APR* was subcloned into pOSB208 using the *Xho*I and *Xba*I sites, respectively. A mixture of donor vector pOSB108-AK and recipient vector pDES200 was then electroporated into *E. coli* strain SW106; *cre* gene expression was induced by adding filter-sterilized arabinose. The recombinant vector was screened using 50 mg/L ampicillin and kanamycin. The vector was digested completely using the homing endonuclease *I-Sce*I to remove the pDESAK vector backbone. For the second round of gene integration, the donor vector pOSB208-APR was recombined with the recipient vector pDESAK following the above procedures to construct the destination vector pDESAK-APR, which contained the *AK* and *APR* genes under the control of the constitutive 35S promoter and the kanamycin resistance gene (*NptII*) as the selection marker.

### Plant material and *Agrobacterium*-mediated transformation

The *Medicago sativa* Chinese cultivar “Baoding” was used in this experiment. The seeds were sown in Murashige and Skoog (MS) agar medium at room temperature with a 16-h light/8-h dark photoperiod. Ten days later, the alfalfa cotyledons were collected, cut into 5-mm square sections, and co-cultivated for 15 min at room temperature with *Agrobacterium tumefaciens* (*EHA105*) inocula containing the transformed vector pDESAK-APR and supplemented with 50 µM acetosyringone, as based on a method described previously [39]. The cotyledon sections were then transferred to Schenk and Hildebrandt (SH) medium [40], [41] in the dark at 25°C for 3–4 days and transferred to SH medium supplemented with 2 mg/L 2,4-dichlorophenoxyacetic acid (2,4-D), 0.025 mg/L kinetin (KT), 250 mg/L carbenicillin (Cb), and 50 mg/L kanamycin (Kan) for approximately 2 weeks. Growth was continued in a callus-developing medium (SH medium supplemented with 2 mg/L 2,4-D, 0.05 mg/L KT, 250 mg/L Cb, and 25 mg/L Kan) for another 2 weeks [39].

The resistant calli were transferred to a differentiation medium (SH medium supplemented with 0.4 mg/L KT, 200 mg/L Cb, and 25 mg/L Kan) for embryonic development and maturation. The plants were regenerated on half-strength MS medium without hormones. To serve as controls, the non-transformed wild-type (WT) plants were also subjected to the same procedures as the transgenic plants except for the co-cultivation with *A. tumefaciens*. Plants with well-defined shoots and roots were transferred to vermiculite and soil (1:1, v/v) in a growth chamber (160×140 mm) at 25°C under a 16-h light/8-h dark photoperiod.

Several shoots from wild-type and overexpressing transgenic plants were vegetatively propagated, and divided to two groups: WT and OE. Through crossing within group, T_1_ seeds were derived and germinated in pots and grown for two months. Leaves were harvested and used for further analysis.

### PCR analysis

Total DNA was extracted from 7-8-week-old leaves of transformed and wild-type alfalfa plants using the cetyltrimethylammonium bromide (CTAB) method according to the manufacturer’s instructions [42]. The primer pairs designed to amplify the 35S promoter and the *AK* and *APR* genes are listed in Table S1. The forward primer was 35S-fr, and the two reverse primers were AK-rv for the *AK* gene and APR-rv for the *APR* gene. The program used to amplify the *AK* gene was as follows: an initial denaturation at 95°C for 5 min, followed by 30 cycles of denaturation at 95°C for 30 s, annealing at 50°C for 45 s, and extension at 72°C for 1 min and 30 s; a final extension was performed at 72°C for 10 min. For the *APR* gene, the same program was used except for the annealing step, which was 56°C for 45 s, with an extension at 72°C for 1 min. The PCR products were separated by electrophoresis through a 1.0% agarose gel and visualized under UV light after staining with ethidium bromide.

### Real-time quantitative PCR (RT-qPCR)

Total RNA was extracted from 7-8-week-old leaves of transformed and wild-type samples using the TRIzol reagent (Invitrogen, Carlsbad, CA, USA) according to the manufacturer’s instructions, and genomic DNA was removed with RNase-free DNaseI (Promega, Madison, WI, USA). A sample (2 µg) of the purified total RNA was used for reverse transcription with M-MLV (Moloney Murine Leukemia Virus) reverse transcriptase (Promega, Madison, WI, USA); the RNA concentration was determined using a spectrophotometer (NanoDrop 1000; Thermo Scientific, Waltham, MA, USA).

*M. sativa* cDNA was synthesized as described above, and the RT-qPCR analysis was performed with a CFX-96 Real-Time System (Bio-Rad, Hercules, CA, USA) using SYBR Premix Ex Taq (TaKaRa, Kyoto, Japan). The primer pairs are listed in Table S1. The PCR cycling conditions were as follows: 95°C for 30 s, followed by 40 cycles of 95°C for 10 s, 59/52°C for 30 s, and 95°C for 10 s. Data were collected during each cycle at 59/52°C by plate reading. A melting curve was generated from 65°C to 95°C to test the amplification specificity, and the relative expression was determined by ΔΔCT. The primers were designed using Beacon Designer 7.0. *M. sativa* actin (GenBank accession number: JQ028730.1) was used as an internal control to normalize the amount of cDNA in the samples. Three independent repetitions were performed using the same conditions in each experiment. All the data were analyzed with Bio-Rad CFX Manager version 1.5.

### Southern blotting

Genomic DNA was isolated from the young leaves of T_0_ transformed and wild-type alfalfa plants using the CTAB method according to the manufacturer’s instructions [42]. Aliquots from each line were digested with the restriction endonuclease *Hind*III, which cleaves outside the *APR* gene, to determine whether the gene was inserted into the plant genome. The digested DNA samples were electrophoresed on 0.8% agarose gels and transferred onto Hybond N+ nylon membranes (Amersham Biosciences, Buckinghamshire, UK) for hybridization. The probe was prepared from an 800-bp *APR* gene fragment by the incorporation of α-^32^P-dCTP using a random-primer DNA labeling kit (Promega, Madison, WI, USA). Standard protocols were used for the Southern blot analysis [42].

### Western blotting

The samples harvested for western blotting included the leaves of transformed and wild-type plants that were approximately 30 days old. The samples were homogenized using a TissueLyser (HerosBio, Beijing, China) and a pestle in a buffer containing 0.25 M NaCl, 1% SDS, 1% mercaptoethanol, and 0.05 M PBS (pH 7.4) at 4°C. The supernatant was collected after 20 min of centrifugation (16,000 x g at 4°C). The protein concentration was measured according to the method of Bradford [43] using the Bio-Rad reagent (Bio-Rad, Hercules, CA, USA) with bovine serum albumin (BSA; Sigma, St. Louis, MO, USA) as a standard. The protein samples (10–20 µg) were fractionated by 10% sodium dodecyl sulfate-polyacrylamide gel electrophoresis (SDS-PAGE) and subsequently transferred to a nitrocellulose (NC) membrane using the Bio-Rad Protein Trans-Blot apparatus (Bio-Rad, Hercules, CA, USA). The membrane was blocked overnight at 4°C in TBST buffer (20 mM Tris [pH 7.5], 150 mM NaCl, and 0.1% Tween 20) containing 5% (v/v) non-fat dry milk; anti-*Pa*APR polyclonal antibodies at a dilution of 1 500 were added, and the membrane was incubated at 25°C for 2 h. The hybridization membrane was then visualized using the NBT/BCIP chromogenic stain.

For antibody production, we synthesized two oligopeptides according to the PaAPR protein sequence: DQSPGTRSQVAVL and QHEREGRWWWEEA. Affinity-purified synthetic oligopeptides coupled to keyhole limpet hemocyanin (Pierce, Thermo Fisher, Rockford, IL, USA) were incubated for 2 hours at 24°C and were used as antigens. Polyclonal antibodies were produced in New Zealand White rabbits at CWBIO Corporation (Beijing, China). The rabbits were sustained on an alfalfa-free diet and prescreened to ensure the absence of endosperm protein cross-reacting antibodies prior to immunization. The resulting polyclonal antibodies were affinity purified with their respective oligopeptide bound to Affigel 15 (Bio-Rad, Hercules, CA, USA), according to standard procedures [44].

For the positive control, His-tagged PaAPR polypeptides were expressed in *E. coli*. The full-length coding sequence of PaAPR was cloned into the pET30a vector (Novagen, Denmark) via the *EcoR*I/*Xho*I sites. The pET30a-APR plasmid was then transformed into *E. coli* (BL21). The His-PaAPR protein was purified by chromatography using Ni-NTA agarose (Invitrogen, Carlsbad, CA, USA) following the manufacturer’s protocol.

### Analysis of total amino acid contents

Leaf samples (approximately 4.5 g) from approximately 16-week-old transgenic and wild-type plants at the early flowering stage were collected and dried in a 70°C drying oven to ensure consistency prior to weighing. The samples were then ground to a fine powder in liquid nitrogen using a TissueLyser (TL2010; HerosBio, Beijing, China) and were kept frozen to heat production, which could inactivate proteins. Each sample was divided into two equal parts. One part was hydrolyzed in 10 mL distilled 6 M HCl at 110°C for 22 h; after drying under vacuum, the sample was homogenized and extracted at 4°C in PBS. The other part was oxidized in peroxyformic acid (30% H_2_O_2_:88% formic acid = 1∶1 [v/v]) for 16 h at 0°C. The reaction was terminated using sodium metabisulfite, and the mixture was hydrolyzed in 6.8 M HCl at 110°C for 22 –24 h. The mixture was neutralized to pH 2.2 using 7.5 M NaOH and homogenized in PBS. After centrifugation, the supernatants were collected and analyzed using an L-8900 amino acid analyzer (Hitachi, Tokyo, Japan) by ion exchange chromatography according to an AOAC method [45]. The acid-hydrolyzed method determined the following amino acids: Asp, Lys, Pro, Tyr, Ala, Ser, Val, Thr, Ile, Leu, Gly, Phe, His, Glu, and Arg; the oxidized-hydrolyzed method determined mainly the sulfur amino acids Cys and Met. Three independent repetitions were performed under the same conditions in each experiment. The data were statistically analyzed by an analysis of variance (ANOVA) using SPSS Statistics 17.0.

### OAS and free amino acid levels

O-acetylserine and free amino acid levels were determined according to a modified protocol [46], [47]. 50 mg leaf tissues were ground to a fine powder in liquid nitrogen using a TissueLyser (TL2010; HerosBio, Beijing, China), and extracted by 400 µl 80% v/v ethanol in 2.5 mM HEPES/KOH (pH 6.2), followed by 400 µl 50% v/v ethanol (pH 6.2) and 200 µl 80% v/v ethanol. Between the extraction steps, the samples were centrifuged for 10 min at 13,000 rpm, 4°C, and the supernatants were collected. The precolumn derivatization was performed by adding the same amount of 0.5% *ortho*-phthalaldehyde (containing 0.4 M borate buffur and 0.1% β-mercaptoethanol, pH 9.5). 20 µl mixture samples were analysed by a LC-20AT HPLC system (SHIMADZU, Kyoto, Japan) (Excitation wavelength 330 nm; emission wavelength 450 nm). Gradient elution was performed by solvent A (50 mM sodium acetate plus 1% tetrahydrofurane, pH 7.0) and solvent B (Methanol) at a flow rate of 0.8 ml/min as follow: 0 min, 80% A, 20% B; 25 min, 12% A, 88% B; 32 min, 80% A, 20% B.

### Analysis of biomass and height of transgenic plants

T_1_ plants and cuttages of T_0_ plants were planted in vermiculite and soil (1∶1 [v/v]) in a growth chamber (240×200 mm) at 25°C under a 16-h-light/8-h-dark photoperiod. At the early flowering stage, the height of each transgenic and wild-type plant was measured, excluding any defects. The aboveground part of every plantlet was collected, dried at room temperature, and weighed; the leaves were then removed and also weighed. Three independent cuttage plants were examined for every line. The data were statistically analyzed by ANOVA using SPSS Statistics 17.0.

## Results

### Construction of the co-expression vector pDESAK-APR and plant transformation

**Figure 1:**
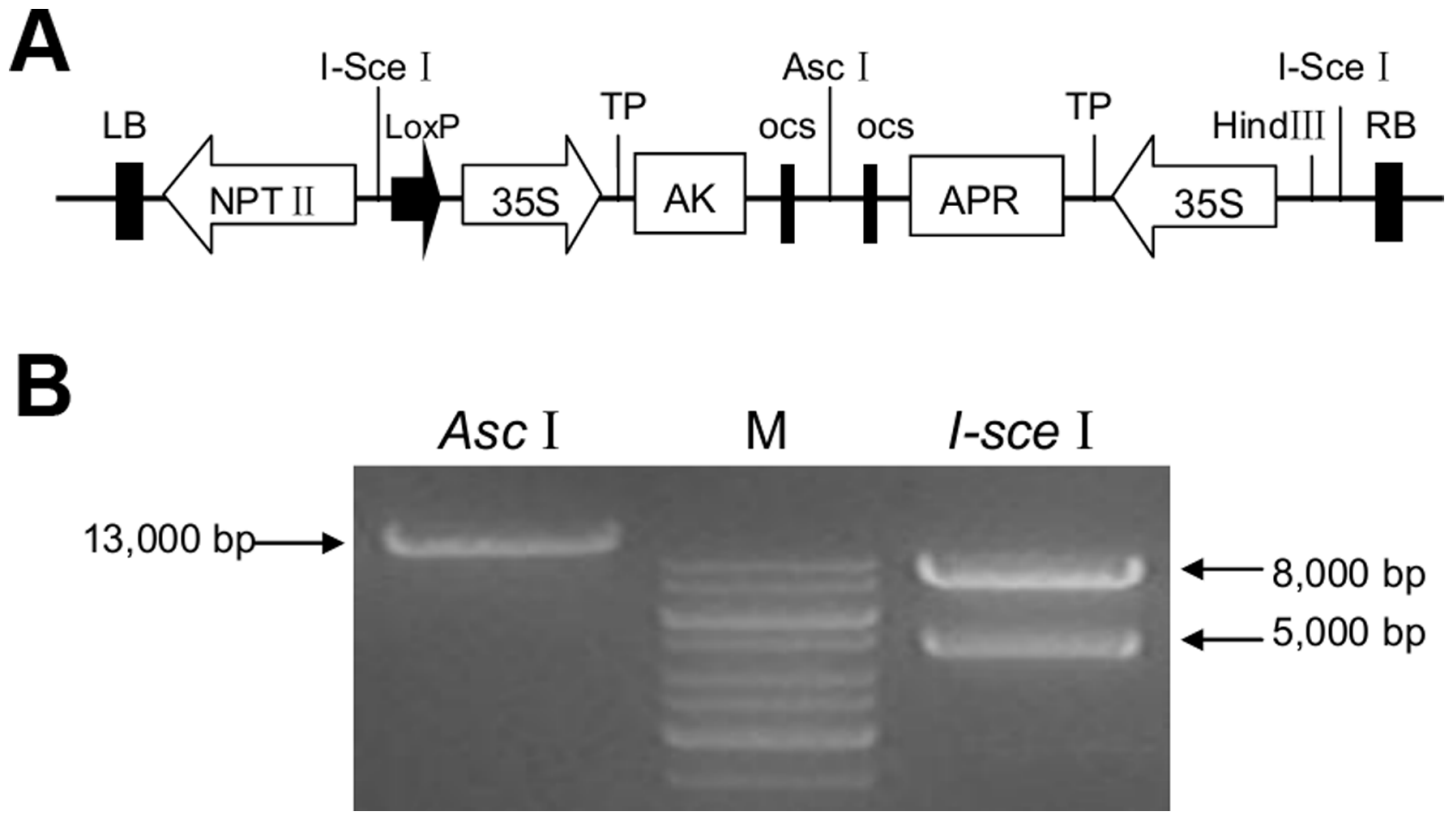
Construction and confirmation of pDESAK-APR. A. Schematic map of the T-DNA region of pDESAK-APR. The *AK* and *APR* genes were fused with the chloroplast transit peptide by restriction digest using *Xho*I and *Xba*I enzymes to construct the intermediate vectors pOSB108-AK and pOSB208-APR. *In vivo* Cre/loxP-mediated recombination via the loxP site to construct the co-expression vector pDESAK-APR. *Hind*III, *Asc*I and *I-sce*I: restriction endonuclease; *NPT*II: kanamycin resistance gene; 35S: cauliflower mosaic virus 35S promoter; TP: chloroplast transit peptide; OCS: octopine synthase terminater; LB and RB: left border and right border of the T-DNA. B. Enzyme digestion analysis of pDESAK-APR. Lane *Asc*I: Vector digested by *Asc*I and Lane *I-sce*I: Vector digested by *I-sce*I. Lane M: molecular mass marker is GeneRuler TM 1 kb DNA ladder (MBI Fermentas, MD, USA). Arrow on the left indicates the expected fragment of 13,000 bp, arrows on the right indicate the expected fragment of 5,000 bp and 8,000 bp.

A co-expression vector was designed based on RMDAP, according to a previous report [38]. The *AK* and *APR* genes were amplified from *E. coli* K12 and *P. aeruginosa* PAO1, respectively. Through site-directed mutagenesis, *AK* was modified to reduce feedback inhibition from downstream metabolites, and the altered *AK* was subcloned into vector pOSB108 containing a chloroplast transit peptide sequence. pOSB108-AK was co-integrated with the recipient vector pDES200 *in vivo* using Cre/loxP-mediated recombination. After another round of Cre/loxP-mediated recombination, the *APR* fragment with a chloroplast transit peptide sequence was also inserted into pDES200. The destination vector pDESAK-APR contained two cauliflower mosaic virus (CaMV) 35S promoters with dual enhancers to drive transcription of the *AK* and *APR* genes in addition to the octopine synthase (OCS) terminator. This vector also contained the kanamycin resistance gene (*NPTII*), which serves as a means for the selection of transformed plants (Fig 1A). The presence of the vector was confirmed by PCR and enzyme digestion (Fig 1B).

Alfalfa cotyledons were transformed with *A. tumefaciens* EHA105 containing pDESAK-APR according to a previous method [39]. After approximately 3–4 months of tissue culture and antibiotic screening, 18 resistant regenerant lines were obtained.

### PCR and Southern blotting confirmed the integration of *AK* and *APR* in the transgenic plants

**Figure 2:**
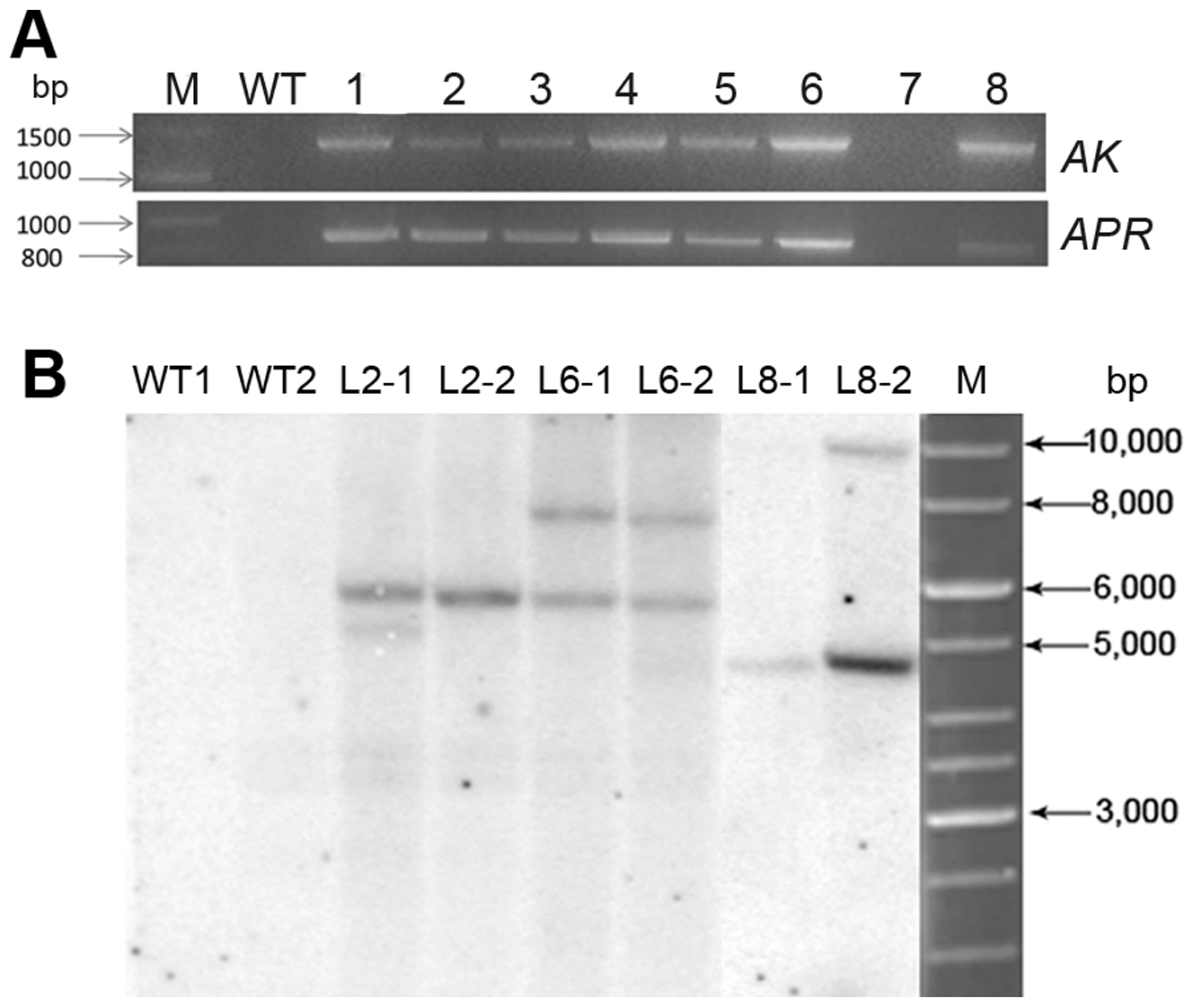
PCR and Southern blot analysis of transgenic alfalfa plants. A. PCR analysis of *AK* and *APR* genes in the transgenic alfalfa plants. Lane WT: wild-type line; Lane1-6: Line1-6 transgenic alfalfa lines; Lane 7: water; Lane 8: pDESAK-APR vector; Lane M: GeneRuler TM 1kb DNA ladder (MBI Fermentas, Maryland, USA). Arrows on the left indicate the standard marker bands: 800 bp, 1,000 bp and 1,500 bp. B. Southern blot analysis of *AK* and *APR* genes in the transgenic alfalfa plants. Lane L2-1,L2-2,L6-1,L6-2,L8-1,L8-2: two replicates of each transgenic line (Line 2, Line 6, Line 8), Lane WT: wild-type line. M: M: GeneRuler TM 1 kb DNA ladder. Arrows on the right indicate the standard marker bands: 3,000 bp, 5,000 bp, 6,000 bp, 8,000 and 10,000 bp.

To confirm the integration of the two cassettes into the genomic DNA of the regenerant plants, PCR was performed using specific primers targeting the CaMV 35S promoter and the *AK* or *APR* gene. 12 resistant lines showed the desired PCR products: 1300 bp (for *AK*) and 800 bp (for *APR*) (only six are listed) (Fig 2A). We randomly selected two plantlets of lines 2, 6, and 8 in addition to two wild-type plants as samples for a Southern blotting analysis. The genomic DNA of the six transgenic plants and two non-transformed control plants was digested with *Hind*III and hybridized to an α-^32^P-dCTP-labeled *APR* gene probe. All six transgenic plants showed hybridization signals, whereas the control did not (Fig 2B). The hybridization signals differed among the transgenic plants, with line 2 showing one hybridization signal and lines 6 and 8 showing two signals. The results of these analyses indicated the presence and stable integration of the *AK* and *APR* genes into the genome of all the transgenic plants examined.

### RT-qPCR confirmed that *AK* and *APR* were expressed at different levels in transgenic plants

**Figure 3:**
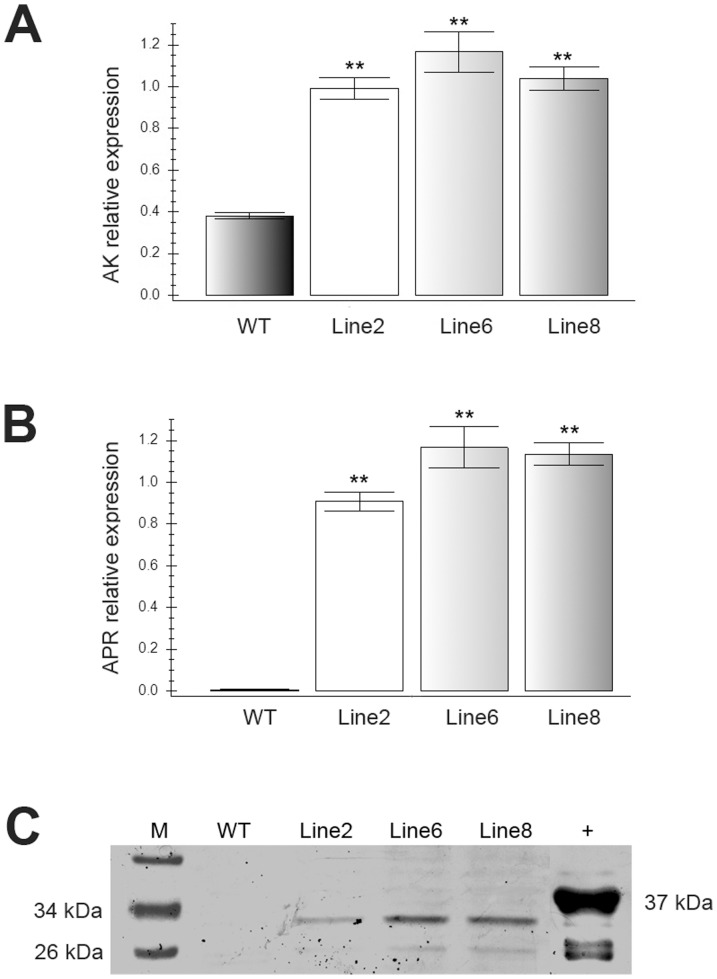
RT-qPCR and Western blot analysis of transgenic alfalfa plants. A. *AK* relative expression level in RT-qPCR analysis, Lane WT: wild-type line; Lane 2,6,8: Line 2,6,8 transgenic alfalfa lines. Each bar represents the mean of three biological replicates±SE. ** represents statistically significant differences (P<0.01). B. *APR* relative expression level in RT-qPCR analysis, Lane WT: wild-type line; Lane 2,6,8: Line 2,6,8 transgenic alfalfa lines. Each bar represents the mean of three biological replicates±SE. ** represents statistically significant differences (P<0.01). C. Western blot assay of expression of APR protein in transgenic alfalfa lines. The total soluble protein extracted from the young leaves of the transgenic lines and non-transformed negative controls. Lane WT: wild-type line, Lane Line2, Line6, Line8: three transgenic lines; Lane +: 6×His-APR fused protein; Lane M: PageRula^r^™ prestained protein ladder (Thermo scientific,USA). 26 kDa and 34 kDa indicate the standard marker bands; 37 kDa indicates the size of 6×His-APR fused protein.

To further accurately assess the transcriptional expression levels of the target genes, an RT-qPCR analysis was performed using lines 2, 6, and 8. Although we designed specific primers for the bacterial *AK* gene, some background expression of the endogenous *AK* was noted because the bacterial *AK* sequence has a high level of homology with alfalfa *AK*. The results revealed that *AK* was upregulated 2-3-fold compared to the non-transformed control (Fig 3A), indicating that *AK* expression in the transgenic plants was enhanced compared to that in the wild-type plants. Because there is no homologous *APR* gene in wild-type alfalfa, the level of *APR* expression could not be quantified in these plants using primers designed for *P. aeruginosa APR*. However, the transformed *APR* gene from *P. aeruginosa* was abundantly expressed in the transgenic plants (Fig 3B). These data showed a trend of upregulation of the overexpressed genes in the transgenic plants compared to the wild-type plants, and these differences were statistically significant.

### Western blotting analysis confirmed the expression of APR in transgenic lines

Because there may be AK homology *in vivo*, we examined the expression of APR protein in the transgenic plants. Total soluble protein was extracted from the transgenic and wild-type plants and analyzed by western blotting. Fig 3C illustrates the presence of a 30-kDa protein band, which is the same size as APR, in lines 2, 6, and 8 using an anti-*Pa*APR polyclonal antibody. The negative control showed no positive signal for the APR protein, and the 6×His-APR fusion protein purified from *E. coli*, which was used as the positive control, showed a protein band of approximately 37 kDa.

### RT-qPCR of *CGS*, *SAT* and *MS* and OAS levels analysis revealed increased sulfur flux

**Figure 4:**
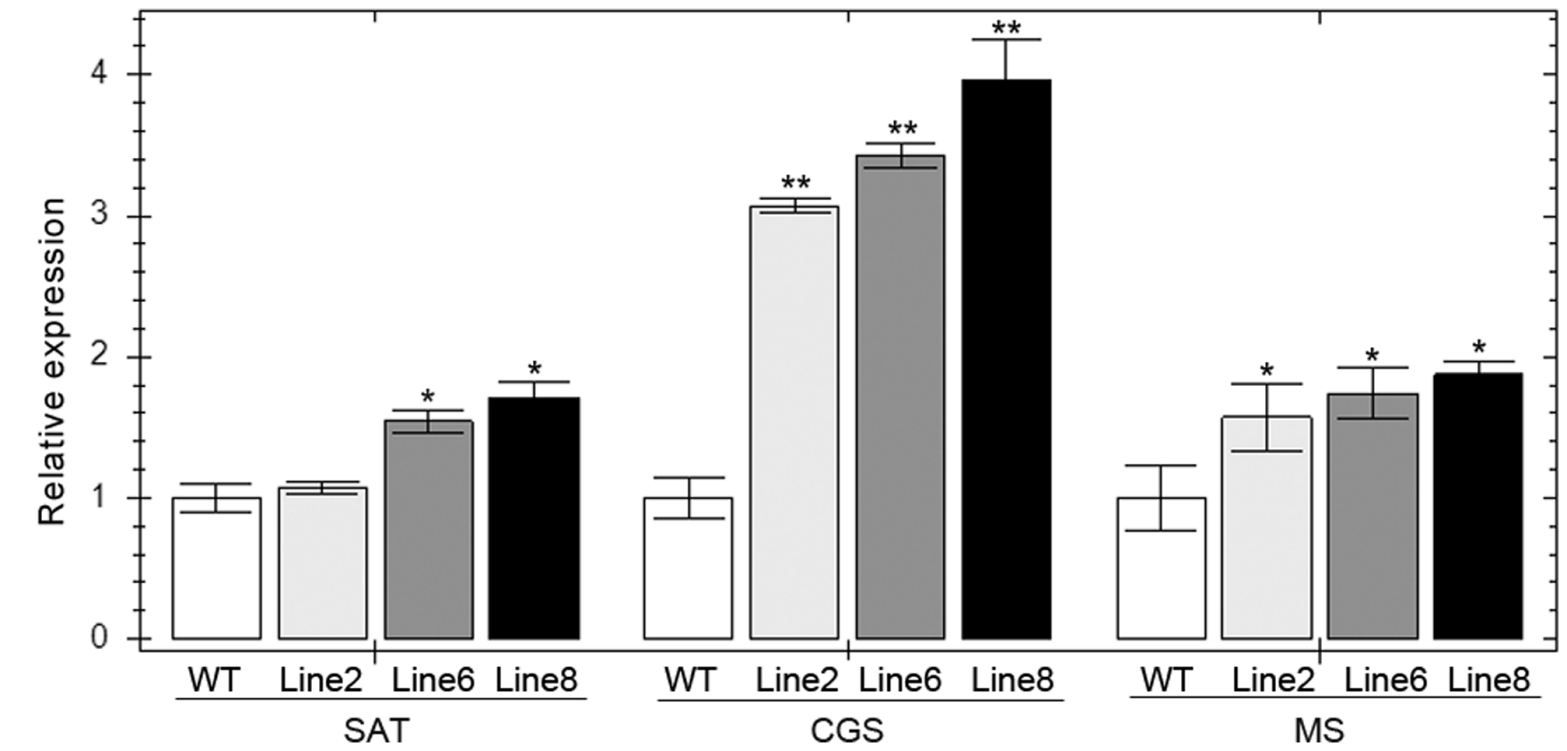
Relative expression levels of SAT, CGS and MS in wild-type and transgenic plants. Lane WT: wild-type line; Lane 2,6,8: Line 2,6,8 transgenic alfalfa lines. SAT: serine acetyltransferase; CGS: cystathionine γ-synthase; MS: methionine synthase. * represents statistically significant differences (P<0.05). ** represents statistically significant differences (P<0.01).

To test the change of sulfur flux in the amino aicds metabolic pathway, some key genes and enzymes were chosen to analyze. Serine acetyltransferase (SAT) catalyses serine to *O*-acetylserine, which is the substrate of cysteine synthesis. CGS catalyses the first step of cysteine to methionine biosynthesis pathway. And, methionine synthase (MS) is the last enzyme of methionine synthesis. As Fig 4 shows, the CGS expression levels in the transgenic plants was enhanced by 3-4 folds significantly compared to WT, and SAT or MS expression was also increased by different amounts. Although increased mRNA levels do not necessarily translate into increased enzyme activity, we cautiously speculated that up-regulation of expression of key enzymes in sulfur assimilation can lead to increased sulfur flux into the direction of SAAs.

**Figure 5:**
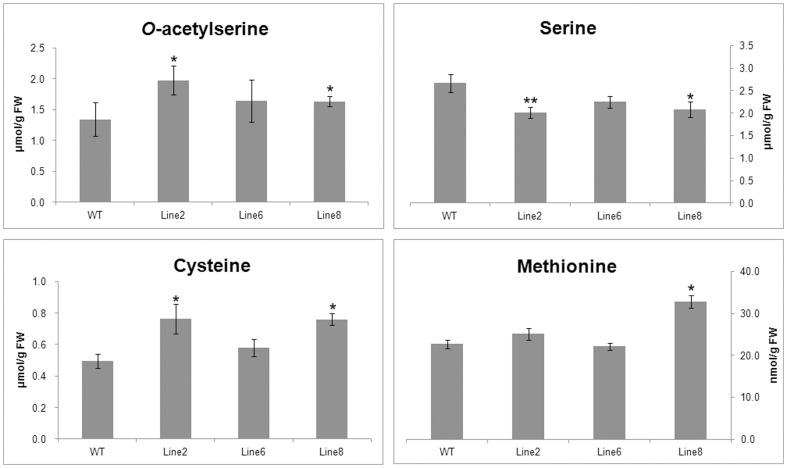
OAS and free amino acid levels in wild-type and transgenic plants. Lane WT: wild-type line; Lane 2,6,8: Line 2,6,8 transgenic alfalfa lines. Each bar represents the mean of three biological replicates±SE. * represents statistically significant differences (P<0.05). ** represents statistically significant differences (P<0.01).

We measured the free *O*-acetylserine (OAS) levels of T_0_ transgenic plants, the results revealed that OAS was increased by 21.0%–47.5% compared to the control (Fig 5). As a signal substance that mediates plant sulfur metabolism [48], [49], increased OAS indicated that sulfur flux of SAAs synthesis pathways was increased in the transgenic plants.

### Overexpression of *AK* and *APR* increased SAAs and altered the amino acid composition

To assess the potential effects of altered sulfur metabolite concentrations resulting from the overexpression of *AK* and *APR* genes, approximately 4.5 g of leaves obtained at the early flowering stage from wild-type plants and 12 transgenic alfalfa lines were collected and ground to a fine powder. Overall, the total amount of 17 different types of amino acids (free and protein-bound amino acids) were quantitated using a Hitachi L-8900 amino acid analyzer by ion exchange chromatography. Of the 12 lines, we chose to examine three (2, 6, and 8), and the results are presented in Table 1.

**Table 1:** Amino acid contents in the leaves of wild-type and transgenic alfalfa plants over-expressing *AK* and *APR.*

Amino acid	Concentration(μmol/g DW)			
	Wild-type	L-2	L-6	L-8
Asp	169.05±12.87	236.66±9.08**	237.42±18.93**	220.89±2.27**
Met	22.79±2.68	30.16±2.01*	32.17±0.67**	36.19±0.67**
Cys	23.10±1.65	27.23±0.83*	23.93±0.81	29.70±0.87*
Lys	84.82±10.26	124.49±0.68**	117.65±2.05**	128.59±2.74**
Pro	178.11±25.20	108.60±0.87**	108.30±0.82**	114.68±0.89**
Glu	211.42±12.31	205.98±0.68	189.99±2.74*	205.98±0.68
Arg	85.53±5.74	82.09±0.57	70.61±1.15**	87.26±0.57
Tyr	51.88±2.76	58.50±0.55	56.84±1.10	41.39±1.10*
Ala	163.86±10.10	178.45±1.12	166.11±1.17	180.70±1.14
Ser	124.64±6.66	114.18±0.95*	108.47±0.95*	122.74±2.85
Val	116.99±6.83	130.66±1.71	130.66±1.71	126.39±3.42
Thr	100.76±6.72	107.47±0.84	102.43±0.82	114.19±0.87*
Ile	86.13±6.10	97.56±1.52*	89.94±0.76	94.51±3.05
Leu	168.45±11.43	181.40±1.52	163.87±0.76	169.97±1.52
Gly	186.42±13.32	198.40±1.33	183.75±1.33	202.40±2.66*
Phe	81.72±4.84	86.56±0.61	81.72±0.61	86.56±1.21
His	38.66±3.22	43.17±0.64	39.30±1.29	45.75±0.65

The data are presented as the means ±SE obtained from three independent measurements.

* represents statistically significant differences (P<0.05). ** represents statistically significant differences (P<0.01). The amounts of amino acids were calculated from dry weight of samples as detected and are given inµmol/g of dry weight (DW) of leaves samples. The total amino acid content did not differ significantly between wild-type and transgenic lines. Three plantlets of each type were analyzed.

**Figure 6:**
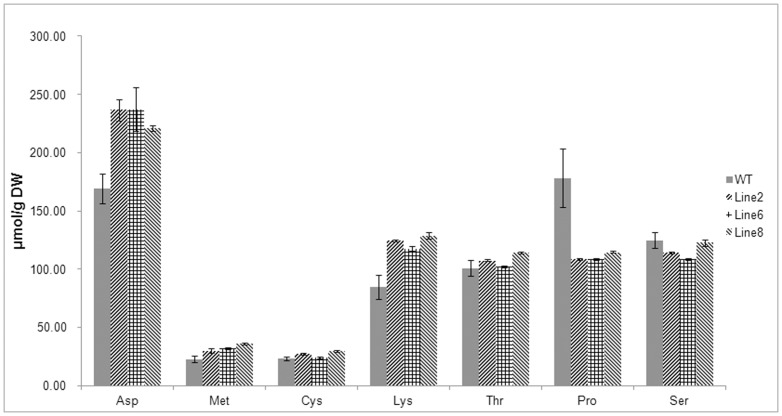
The content of crucial amino acids in leaves of wild-type and transgenic plants. Extracts were obtained from the leaves of 16-week-old non-transformed plants and transgenic plants co-expressing bacterial *AK* and *APR*. Seven kinds of crucial amino acids were list. The amounts of amino acids were calculated from dry weight of samples as detected by Hitachi L-8900 and are given inµmol/g DW. The data presented represent the mean and the bars represent ±SE of three plants per line.

Cys and Met, which are the most important sulfur amino acids, were increased by 17.8% and 32.5% in line 2 and 28.6% and 58.8% in line 8, respectively. The Met level in line 6 increased by 41.2%; however, the Cys level in line 6 showed no significant difference (Fig 6). The results revealed large increases of Asp and Lys of approximately 40% and 45%, whereas Pro significantly decreased by an average of approximately 40% compared to the wild-type plants. The levels of EAAs, such as Asp, Lys, Met, and Cys, in the plants expressing both genes were all substantially increased though to different extents; these increases were accompanied by a significant reduction in the Pro, Tyr, and Arg levels, whereas Gln, Ser, and Gly were slightly decreased relative to the control. The levels of other amino acids did not significantly differ from those of the non-transformed plants (Table 1).

To test whether the higher levels of SAAs resulted from free or bound amino acids, we measured free Ser, Cys and Met levels by HPLC. The results revealed the similar pattern to the total amino acids (Fig 5), free Ser was decreased by 20.6%, but free Cys and Met were increased by 41.7% and 17.6% respectively. As free amino acids constituted only a small proportion of total amino acids, the protein-bound levels would be of more importance in the increasing of SAAs.

### Analysis of T_1_ plants

PCR, RT-qPCR and Western blot analysis of T_1_ transgenic plants were presented in Figure S1. PCR showed T_1_ lines: BD1-BD8 all reserved *AK* and *APR* genes (Fig S1A). T1-BD1, T1-BD5 and T1-BD8 were chosen to be used for further analysis. RT-qPCR revealed *AK* and *APR* expression levels was increased significantly in three transgenic plants compared to wild-type plant (Fig S1B), and Western blotting showed AK and APR proteins were expressed in T_1_ transgenic plants, and the control did not (Fig S1C).

Total and free amino acids levels of T_1_ transgenic plants indicated a similar results to T_0_ plants (Fig S2). Total Cys was increased by 10.7–21.0% and Met was increased by 10.1–40.7%. Free OAS, Cys and Met levels were also increased by 169.2%, 47.4% and 49.8% averagely compared to the wild-type plants. *AK* and *APR* were stable inherited and expressed in T_1_ progeny.

### Analysis of total amino acids and forage biomass revealed that the transgenic events had no significant influence on the growth of alfalfa

**Figure 7:**
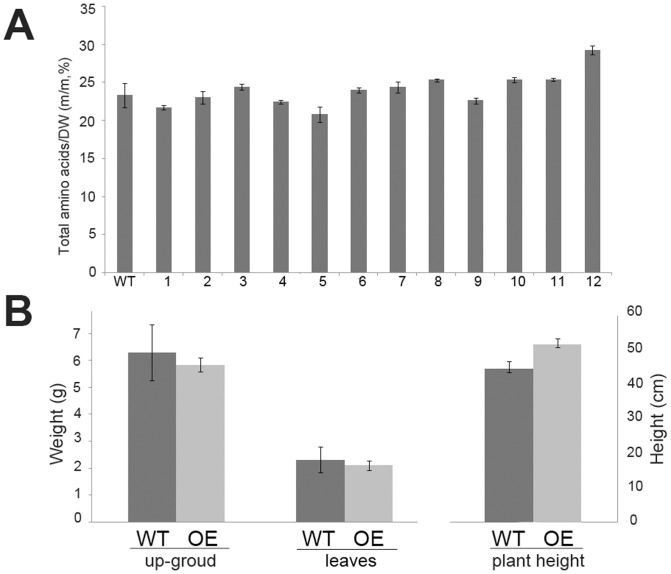
Analysis of total amino acids and forage products in wild-type and transgenic plants. A. Total amino acid content in leaves of transgenic and wild-type plants. m/m, % represented mass ratio of total amino acids from dry weight; WT: wild-type plant; 1–12: 12 lines of transgenic alfalfa plants; the bar represented ±SE. B. Weight of up-ground part and leaves products and height of wild-type and transgenic plants. WT : wild-type plant; OE: the average number of 12 lines of transgenic alfalfa plants, the bar represented ±SE.

Changes in the amounts of certain amino acids might affect the balance of other amino acids and metabolism products or even impact the growth and phenotype of the plant. And, to test whether the higher levels of SAAs resulted from increase of total protein in transgenic plants, we measured the total amino acid content in the leaves of each transgenic and wild-type plant. As presented in Fig 7A, the data represent the mass ratio of the total amino acids from dry matter biomass (Table S2) and indicated no significant differences in the growth parameters between the transgenic and wild-type plants.

To determine the effect of transgenic events on the growth of alfalfa, we measured the height of the plants and the weight of the leaves and the aboveground parts (Fig 7B); the data are presented in Table S3. Based on the statistical analysis, the results indicated that the transgenic plants were slightly taller than the wild-type plants, though the weights of the leaves and aboveground parts of the plants were not notably different. And, T_1_ transgenic plants also revealed the similar phenotype (data not shown) to T_0_ transgenic plants. The transgenic nature of the plants also did not have any obvious negative effect on the phenotype. Overall, the transgenic events and the change in amino acid composition did not appear to have a significant influence on the growth or development of alfalfa.

## Discussion

**Figure 8:**
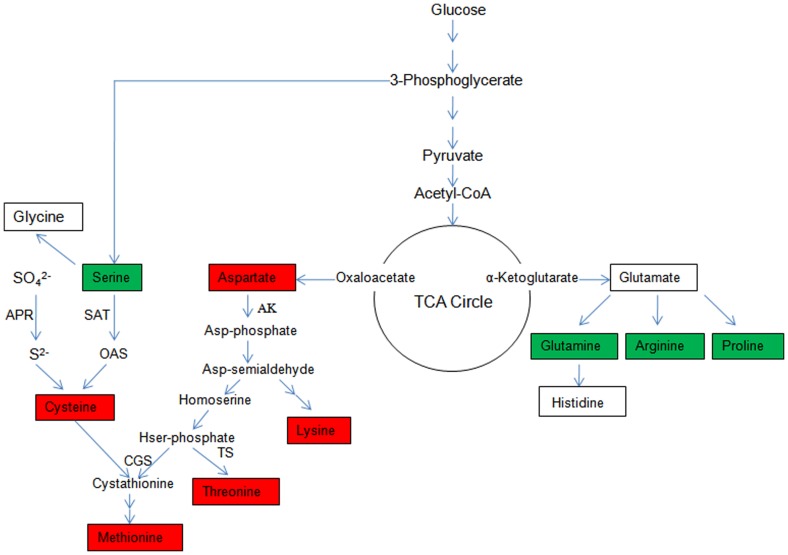
Amino acid metabolic pathways and its integration into the TCA cycle. Red indicates increased amino acids; Green indicates decreased amino acids.

Sulfur amino acids are valuable for pasture and livestock production. Hence, an enhancement of the biosynthesis and accumulation of SAAs via genetic modification has become an important issue. There are two main ways to enhance the expression of SAAs according to the amino acid metabolic pathway (Fig 8). One method is dependent on the bioconversion of other amino acids into sulfur amino acids; for example, overexpressing bacterial AK resulted in the accumulation of Thr in the leaves of transgenic tobacco [18], [19], alfalfa [19], and *Arabidopsis* [50] and Met in the seeds of tobacco [20]. The other method is related to the assimilation of sulfate. Each method has some flaws that restrict its application, including the feedback inhibition of enzymes by other metabolites and the control of key pathways by other metabolic pathways. Therefore, we chose two key enzymes (AK and APR) in different metabolic pathways that could function cooperatively to increase SAAs. AKIII from *E. coli* was chosen because bacterial enzymes retain the same function as the natural plant enzymes while being insensitive to the feedback inhibition of other metabolites. Furthermore, the modification of *AKIII* genes through site-directed mutagenesis could reduce the feedback inhibition from Lys [51]. *Pa*APR is more stable than plant APR [33], [52] and can utilize plant thioredoxins [28], [30], [31]. We constructed an expression vector containing both *AK* and *APR* gene cassettes to transform alfalfa, allowing the T-DNA region of the co-expressing vector to be integrated into the genome of the transgenic plants and be stably inherited by the progeny.

Overexpression of SAT could resulted in dramatic increases in the concentrations of OAS and free cysteine[53]–[55]. CGS from Arabidopsis (AtCGS) was transferred into different species, such as Arabidopsis [56], tobacco [57], potato [58] and alfalfa [7], the results all increased methionine levels by different amounts. And, OAS has been considered to be a signal that mediates plant sulfur metabolism [48], [49] and protein composition [59]. Our results supported previous studies, and proved enhanced sulfur flux in the transgenic plants.

We measured the free and total amino acids in the wild-type and transgenic plants. As an elevated level of free amino acids can promote itself incorporate into proteins[19], [60], [61], and free amino acids constituted only a very small proportion of total amino acids, we focused on the total amino acid levels. The results revealed that the transgenic plants not only produced higher levels of SAAs but also exhibited enhanced contents of other EAAs, such as Asp and Lys. However, different transgenic lines differed in their contents of these amino acids. For example, line 8 had the highest levels of Met, Cys, and Lys, whereas line 6 showed the highest levels of Asp. This result may be explained by the fact that the number of insertion events varied between the different transgenic lines, as based on the results of Southern blotting, which could cause slight differences among the plants. Although our results confirmed that the co-expression of *AK* and *APR* led to an increase in SAAs and a decrease in some other amino acids, the amount of total amino acids did not change significantly. Additionally, the transgenic plants showed no signs of abnormal phenotypes, and their growth was normal. The *Arabidopsis* progeny obtained from crossing an AtLKR/SDH knockout mutant with a DHPS overexpression line exhibited severe abnormal phenotypes and overaccumulated Lys in their leaves (an 8-fold increase compared to the parental lines) [62]. This result indicates that plants have a tolerance limit with respect to changes in amino acid levels, and there may also be a balance between substance and energy in plants that acts systematically to regulate growth and development.

We measured the contents of 17 different types of amino acids, and our results indicated a significant metabolic connection among those involved in the aspartate family pathway, the glutamine family pathway, and the tricarboxylic acid (TCA) cycle (Fig 8). Asp is synthesized by oxaloacetate, and Cys and Met (of the Asp family) increased by approximately 30% and 60%, respectively, compared to their levels in the wild-type plants, indicating that the level of substrate (oxaloacetate) must be increased. In contrast, Ser and Pro (of the Glu family) decreased by approximately 10% and 20%. It is known that the TCA cycle is an energy production and transmission pathway but is also a connection point between different amino acid metabolism pathways. Glu is synthesized from α-oxoglutarate, which, along with oxaloacetate, is one of the most important metabolites of the TCA cycle. In the TCA cycle, increased Asp family substances are inclined to flow from α-oxoglutarate to oxaloacetate, resulting in the upregulation of Asp family amino acids and downregulation of Glu family amino acids. Our conclusion may be explained by the fact that overexpressing the *AK* gene produced more Asp-phosphate, the substrate for Met, Thr, and Lys syntheses. It was previously reported that *AK* overexpression can increase the level of Thr in transgenic alfalfa [19]. In addition, overexpressing the *APR* gene could increase S^2–^ to enhance cysteine levels. The synergism of these two genes also improved methionine synthesis (Fig 8). In addition, a significant reduction in the levels of TCA cycle-associated metabolites in *Arabidopsis* seeds, with increased lysine levels, was also reported [63]. Thus, there is a dynamic balance between amino acid metabolism and the TCA cycle.

It was previously reported in transgenic alfalfa that an increase in cysteine was likely at the expense of serine, the carbon skeleton donor of cysteine; in fact, the serine level in the soluble protein fraction was found to be reduced by approximately 2-fold [7]. Our results also supported this conclusion. In studies examining serine family amino acid biosynthetic pathways, no matter free or total amino acids, the results often indicate that the level of serine declines while the cysteine level is greatly increased. Indeed, this may be the case because the overexpression of *APR* leads to an increase in S^2–^, and OAS reacts with H_2_S to produce more cysteine, which is accompanied by a decrease in serine.

In summary, the approach we present here both provides a new method for the regulation of SAAs biosynthesis through the co-expression of *AK* and *APR* and also allows for the development of a new genetically modified crop with a greater nutritional value.

## Supporting Information

Figure S1 Molecular analysis of T_1_ wild-type and transgenic plants. A. PCR analysis of *AK* and *APR* genes in T_1_ transgenic alfalfa plants. Lane WT: wild-type line; Lane1-8: T_1_-BD1-8 transgenic alfalfa lines. +: positive control(vector). B. *AK* and *APR* relative expression levels of T_1_ transgenic alfalfa plants in RT-qPCR analysis. Lane WT: wild-type line; Lane T1-BD1,5,8: T_1_ transgenic alfalfa lines. Each bar represents the mean of three biological replicates±SE. ** represents statistically significant differences (P<0.01). C. Western blot assay of expression of APR protein in T_1_ transgenic alfalfa lines. Lane WT: wild-type line; Lane T1-BD1,5,8: T_1_ transgenic alfalfa lines; +: 6×His-APR fused protein; Lane M: PageRula^r^™ prestained protein ladder (Thermo scientific,USA). 26 kDa and 34 kDa indicate the standard marker bands.
(DOCX)

Figure S2 OAS and amino acid levels in T_1_ wild-type and transgenic plants. A. Total amino acid levels in T_1_ wild-type and transgenic plants. B. OAS and free amino acid levels in T_1_ wild-type and transgenic plants. WT: wild-type line; T1-BD1,5,8: T_1_ transgenic alfalfa lines. DW: Dry Weight; FW: Fresh Weight. * represents statistically significant differences (P<0.05). ** represents statistically significant differences (P<0.01).
(DOCX)

Table S1 Sequence of oligonucelotides used in experiments. fr indicates forward primer and rv indicated reverse primer.
(DOCX)

Table S2 Contents of total amino acids from the dry weight in wild-type (WT) and transgenic alfalfa overexpressing *AK* and *APR* (Line 1-12).
(DOCX)

Table S3 The height and the weight of the up-ground products of wild-type (WT) and transgenic alfalfa overexpressing *AK* and *APR* (OE).
(DOCX)
