# Correction: Co-Expression of Bacterial Aspartate Kinase and Adenylylsulfate Reductase Genes Substantially Increases Sulfur Amino Acid Levels in Transgenic Alfalfa (*Medicago sativa* L.)

**DOI:** 10.1371/journal.pone.0105182

**Published:** 2014-08-01

**Authors:** The PLOS ONE Staff

Figure S1A is incorrect. Please see the corrected Figure S1 below.

## Supporting Information

Figure S1 Molecular analysis of T_1_ wild-type and transgenic plants. A. PCR analysis of *AK* and *APR* genes in T_1_ transgenic alfalfa plants. Lane WT: wild-type line; Lane1-8: T_1_-BD1-8 transgenic alfalfa lines. +: positive control(vector). B. *AK* and *APR* relative expression levels of T_1_ transgenic alfalfa plants in RT-qPCR analysis. Lane WT: wild-type line; Lane T1-BD1,5,8: T_1_ transgenic alfalfa lines. Each bar represents the mean of three biological replicates±SE. ** represents statistically significant differences (P<0.01). C. Western blot assay of expression of APR protein in T_1_ transgenic alfalfa lines. Lane WT: wild-type line; Lane T1-BD1,5,8: T_1_ transgenic alfalfa lines; +: 6×His-APR fused protein; Lane M: PageRula^r^™ prestained protein ladder (Thermo scientific,USA). 26 kDa and 34 kDa indicate the standard marker bands.
(TIF)
