# Supplementary material for: Co-Expression of Bacterial Aspartate Kinase and Adenylylsulfate Reductase Genes Substantially Increases Sulfur Amino Acid Levels in Transgenic Alfalfa (Medicago sativa L.)
Source: PLoS One. 2014 Feb 10;9(2):e88310. doi: 10.1371/journal.pone.0088310 (PMC3919742; doi:10.1371/journal.pone.0088310)
Supplement: Figure S1 — Molecular analysis of T1 wild-type and transgenic plants. A. PCR analysis of AK and APR genes in T1 transgenic alfalfa plants. Lane WT: wild-type line; Lane1-8: T1-BD1-8 transgenic alfalfa lines. +: positive control(vector). B. AK and APR relative expression levels of T1 transgenic alfalfa plants in RT-qPCR analysis. Lane WT: wild-type line; Lane T1-BD1,5,8: T1 transgenic alfalfa lines. Each bar represents the mean of three biological replicates±SE. ** represents statistically significant differences (P<0.01). C. Western blot assay of expression of APR protein in T1 transgenic alfalfa lines. Lane WT: wild-type line; Lane T1-BD1,5,8: T1 transgenic alfalfa lines; +: 6×His-APR fused protein; Lane M: PageRular™ prestained protein ladder (Thermo scientific,USA). 26 kDa and 34 kDa indicate the standard marker bands. (DOCX) [file pone.0088310.s001.docx]

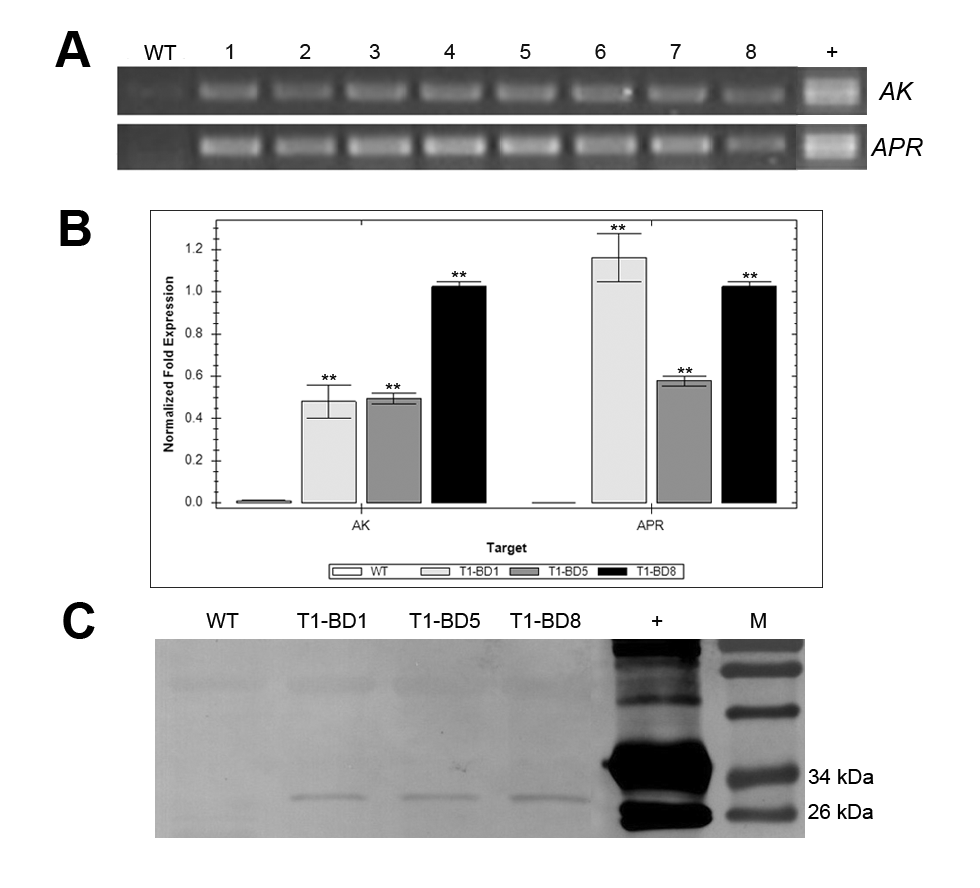


**Fig S1 Molecular analysis of T_1_ wild-type and transgenic plants.**

1. PCR analysis of *AK* and *APR* genes in T_1_ transgenic alfalfa plants.

Lane WT: wild-type line; Lane1-8: T_1_-BD1-8 transgenic alfalfa lines. +: positive control(vector).

1. *AK* and *APR* relative expression levels of T_1_ transgenic alfalfa plants in RT-qPCR analysis.

Lane WT: wild-type line; Lane T1-BD1,5,8: T_1_ transgenic alfalfa lines. Each bar represents the mean of three biological replicates±SE.

** represents statistically significant differences (P<0.01).

C. Western blot assay of expression of APR protein in T_1_ transgenic alfalfa lines.

Lane WT: wild-type line; Lane T1-BD1,5,8: T_1_ transgenic alfalfa lines;

+: 6×His-APR fused protein; Lane M: PageRula^rTM^ prestained protein ladder (Thermo scientific,USA). 26 kDa and 34 kDa indicate the standard marker bands.
