# Supplementary material for: Co-Expression of Bacterial Aspartate Kinase and Adenylylsulfate Reductase Genes Substantially Increases Sulfur Amino Acid Levels in Transgenic Alfalfa (Medicago sativa L.)
Source: PLoS One. 2014 Feb 10;9(2):e88310. doi: 10.1371/journal.pone.0088310 (PMC3919742; doi:10.1371/journal.pone.0088310)
Supplement: Figure S2 — OAS and amino acid levels in T1 wild-type and transgenic plants. A. Total amino acid levels in T1 wild-type and transgenic plants. B. OAS and free amino acid levels in T1 wild-type and transgenic plants. WT: wild-type line; T1-BD1,5,8: T1 transgenic alfalfa lines. DW: Dry Weight; FW: Fresh Weight. * represents statistically significant differences (P<0.05). ** represents statistically significant differences (P<0.01). (DOCX) [file pone.0088310.s002.docx]

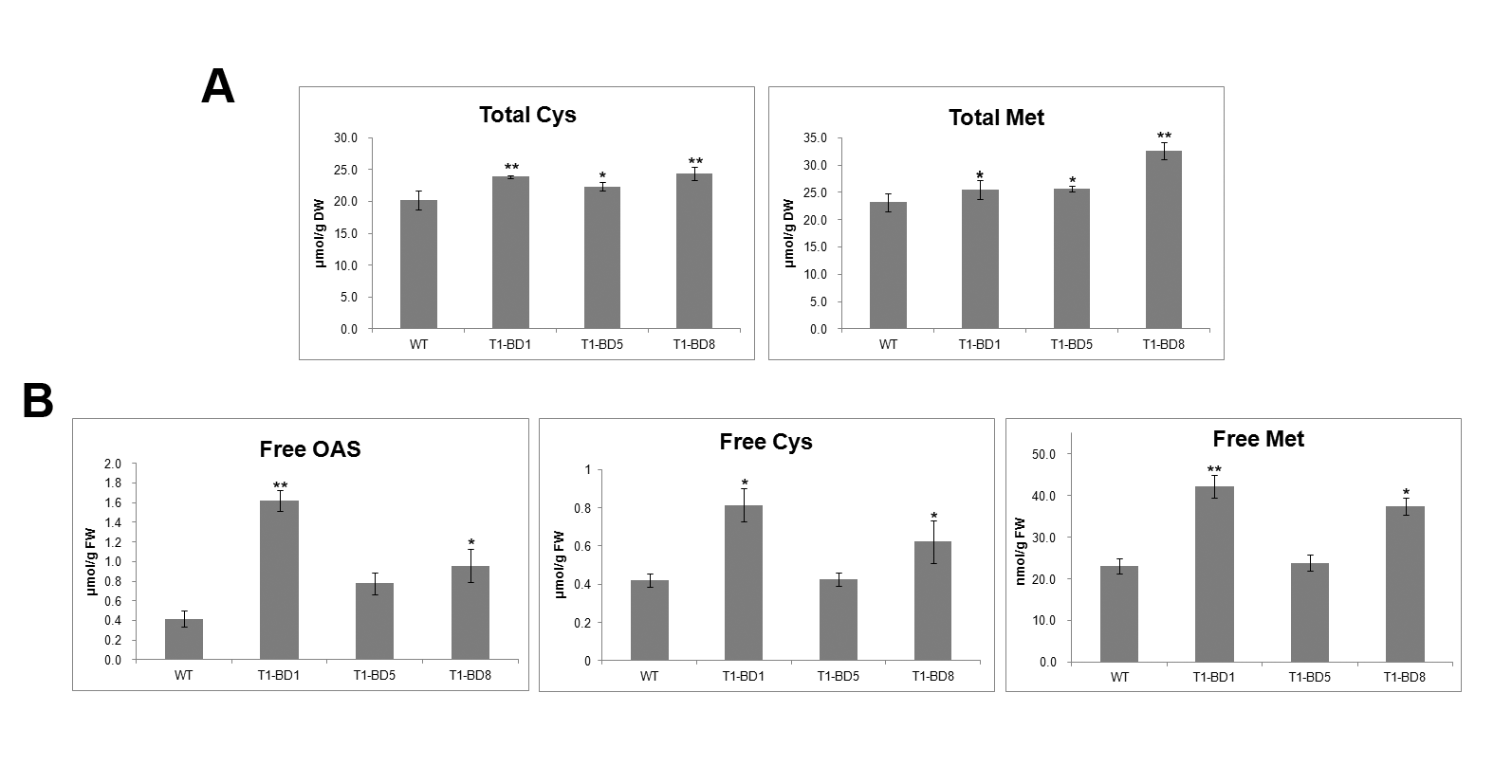


**Fig S2 OAS and amino acid levels in T_1_ wild-type and transgenic plants.**

A. Total amino acid levels in T_1_ wild-type and transgenic plants.

B. OAS and free amino acid levels in T_1_ wild-type and transgenic plants.

WT: wild-type line; T1-BD1,5,8: T_1_ transgenic alfalfa lines. DW: Dry Weight; FW: Fresh Weight. * represents statistically significant differences (P<0.05). ** represents statistically significant differences (P<0.01).
