# Supplementary material for: Co-Expression of Bacterial Aspartate Kinase and Adenylylsulfate Reductase Genes Substantially Increases Sulfur Amino Acid Levels in Transgenic Alfalfa (Medicago sativa L.)
Source: PLoS One. 2014 Feb 10;9(2):e88310. doi: 10.1371/journal.pone.0088310 (PMC3919742; doi:10.1371/journal.pone.0088310)
Supplement: Table S1 — Sequence of oligonucelotides used in experiments. fr indicates forward primer and rv indicated reverse primer. (DOCX) [file pone.0088310.s003.docx]

**Table S1** **Sequence of oligonucelotides used in experiments.** fr indicates forward primer and rv indicated reverse primer.

| Primer name | Oligonucleotide sequence |
| --- | --- |
| AK-fr | 5’-CTCGAGTCTGAAATTGTTGTCTCCAA-3’ |
| AK-rv | 5’-TCTAGATTACTCAAACAAATTACTAT-3’ |
| AKmu-fr | 5’-CGTCAGAAGTGAGCGTGGCATTAATC CTTGATACCA-3’ |
| AKmu-rv | 5’-TGGTATCAAGGATTAATGCCACGCTCACTTCTGACG-3’ |
| APR-fr | 5’-CCGCTCGAGCTGCCCTTTGCTACCATTCC-3’ |
| APR-rv | 5’-GCTCTAGACATCAGGCCTTGCTGATCAGGT-3’ |
| 35S-fr | 5’-GACGCACAATCCCACTATCC-3’ |
| *Ma*actin-fr | 5’-CCCACTGGATGTCTGTAGGTT-3’ |
| *Ma*actin-rv | 5’- AGAATTAAGTAGCAGCGCAAA-3’ |
| AKrt-fr | 5’-CTTCTGCTGGTATCACTAA-3’ |
| AKrt-rv | 5’- GTTCAATCTCTTCACGGATA-3’ |
| APRrt-fr | 5’-GTCGTCCTGGTAGACATG-3’ |
| APRrt-rv | 5’-TGGTCGATGAAACGATAG-3’ |
| SATrt-fr | 5’-CAGGAAGAAGCTAAAGTT-3’ |
| SATrt-rv | 5’-CAGATTCCAAAATTCTAACA-3’ |
| CGSrt-fr | 5’-CATCTTGTGACTACTACTG-3’ |
| CGSrt-rv | 5’-CATCTCAGGAATGGATTG-3’ |
| MSrt-fr | 5’-TCATCAGTTACAAGCATT-3’ |
| MSrt-rv | 5’-ACAAGGTCAAATCCATAT-3’ |
