# Supplementary material for: Co-Expression of Bacterial Aspartate Kinase and Adenylylsulfate Reductase Genes Substantially Increases Sulfur Amino Acid Levels in Transgenic Alfalfa (Medicago sativa L.)
Source: PLoS One. 2014 Feb 10;9(2):e88310. doi: 10.1371/journal.pone.0088310 (PMC3919742; doi:10.1371/journal.pone.0088310)
Supplement: Table S2 — Contents of total amino acids from the dry weight in wild-type (WT) and transgenic alfalfa overexpressing AK and APR (Line 1-12). (DOCX) [file pone.0088310.s004.docx]

**Table S2 Contents of total amino acids from the dry weight in wild-type (WT) and transgenic alfalfa overexpressing *AK* and *APR* (Line 1-12)*.***

| Plants | Total amino acids of dry matter (m/m,%) |
| --- | --- |
| WT | 23.27±1.59 |
| Line 1 | 21.69±0.31 |
| Line 2 | 22.99±0.82 |
| Line 3 | 24.39±0.43 |
| Line 4 | 22.40±0.27 |
| Line 5 | 20.77±1.00 |
| Line 6 | 23.97±0.37 |
| Line 7 | 24.35±0.69 |
| Line 8 | 25.29±0.19 |
| Line 9 | 22.58±0.38 |
| Line 10 | 25.33±0.36 |
| Line 11 | 25.38±0.19 |
| Line 12 | 29.23±0.55 |

The data are presented as the means±SE obtained from three independent measurements.
