# Supplementary material for: Co-Expression of Bacterial Aspartate Kinase and Adenylylsulfate Reductase Genes Substantially Increases Sulfur Amino Acid Levels in Transgenic Alfalfa (Medicago sativa L.)
Source: PLoS One. 2014 Feb 10;9(2):e88310. doi: 10.1371/journal.pone.0088310 (PMC3919742; doi:10.1371/journal.pone.0088310)
Supplement: Table S3 — The height and the weight of the up-ground products of wild-type (WT) and transgenic alfalfa overexpressing AK and APR (OE). (DOCX) [file pone.0088310.s005.docx]

**Table S3** **The height and the weight of the up-ground products of wild-type (WT) and transgenic alfalfa overexpressing *AK* and *APR* (OE)**

The data are presented as the means±SE obtained from three independent measurements.

| Plants | Plant Height(cm) | Up-ground Products(g) | Leaves Products(g) |
| --- | --- | --- | --- |
| WT-1 | 46.1 | 7.35 | 2.59 |
| WT-2 | 42.8 | 5.25 | 1.44 |
| WT-3 | 43.4 | 6.50 | 2.92 |
| WT | 44.1±1.2 | 6.20±0.95 | 2.32±0.58 |
| L2-1 | 52.3 | 6.25 | 2.61 |
| L2-2 | 47.7 | 6.55 | 2.84 |
| L2-3 | 50.6 | 5.20 | 1.33 |
| L6-1 | 59.3 | 5.10 | 2.14 |
| L6-2 | 52.8 | 6.00 | 1.92 |
| L6-3 | 48.6 | 6.15 | 2.16 |
| L8-1 | 45.2 | 4.80 | 1.62 |
| L8-2 | 51.4 | 5.76 | 2.03 |
| L8-3 | 52.1 | 6.70 | 2.15 |
| OE | 51.1±1.4 | 5.83±0.24 | 2.09±0.16 |
