# Supplementary figures and images for: Correction: Co-Expression of Bacterial Aspartate Kinase and Adenylylsulfate Reductase Genes Substantially Increases Sulfur Amino Acid Levels in Transgenic Alfalfa (Medicago sativa L.)
Source: PLoS One. 2014 Aug 1;9(8):e105182. doi: 10.1371/journal.pone.0105182 (PMC4118966; doi:10.1371/journal.pone.0105182)

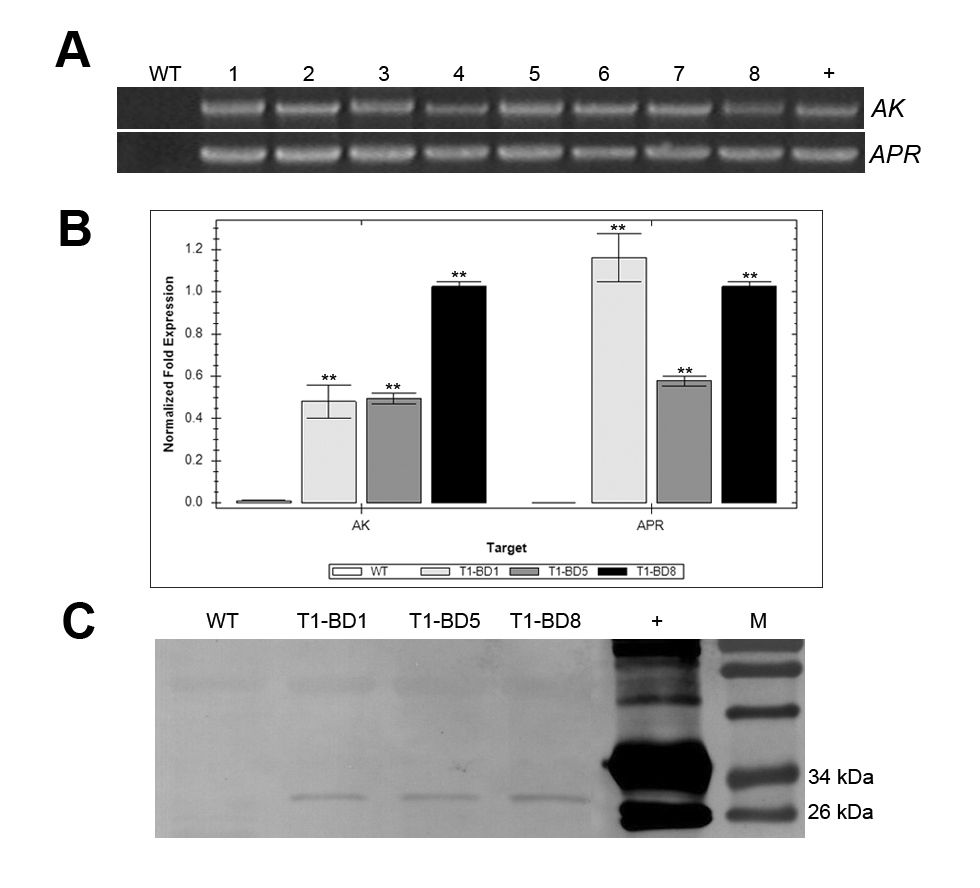

Supplement: Figure S1 — Molecular analysis of T1 wild-type and transgenic plants. A. PCR analysis of AK and APR genes in T1 transgenic alfalfa plants. Lane WT: wild-type line; Lane1-8: T1-BD1-8 transgenic alfalfa lines. +: positive control(vector). B. AK and APR relative expression levels of T1 transgenic alfalfa plants in RT-qPCR analysis. Lane WT: wild-type line; Lane T1-BD1,5,8: T1 transgenic alfalfa lines. Each bar represents the mean of three biological replicates±SE. ** represents statistically significant differences (P<0.01). C. Western blot assay of expression of APR protein in T1 transgenic alfalfa lines. Lane WT: wild-type line; Lane T1-BD1,5,8: T1 transgenic alfalfa lines; +: 6×His-APR fused protein; Lane M: PageRular™ prestained protein ladder (Thermo scientific,USA). 26 kDa and 34 kDa indicate the standard marker bands. (TIF) [file pone.0105182.s001.tif]
